# La maladie de Wilson: à propos d'un cas familial

**DOI:** 10.11604/pamj.2014.18.270.5049

**Published:** 2014-08-02

**Authors:** Yassine Mouzari, Ryme Abdelkhalek, Fouad El Asri, Karim Reda, Abedelbarre Oubaaz

**Affiliations:** 1 Service d'Ophtalmologie de l'Hôpital Militaire Mohamed V, Rabat, Maroc

**Keywords:** Maladie de Wilson, anneau de Keyser-Fleischer, dépistage, Wilson disease, Keyser-Fleischer ring, screening

## Abstract

La maladie de Wilson est une maladie rare autosomique récessive due à une diminution de l’élimination du cuivre dans la bile et son accumulation toxique dans les organes en particulier le cerveau, le foie, la cornée et le rein d'où son hétérogénéité clinique. Les manifestations ophtalmologiques représentent des critères diagnostiques importants. Le traitement précoce permet une réversibilité des déficits; non traitée la maladie de Wilson est létale. Nous rapportons un cas familial de la maladie de Wilson: deux membres d'une fratrie issus d'un mariage consanguin étaient atteints de la maladie de Wilson dans ses trois formes cliniques: hépatique, neurologique et psychiatrique. Les manifestations ophtalmologiques de la maladie de Wilson sont l'anneau de Keyser Fleischer et la cataracte en tournesol, l'atteinte hépatique se manifeste par une hépatite chronique et une cirrhose, la symptomatologie neurologique et psychiatrique est variée; on retrouve à l'IRM une atteinte prédominante aux noyaux gris centraux. Le diagnostic positif de la maladie de Wilson est fait sur la triade: présence de l'anneau de Keyser Fleischer, céruloplasmine sanguine basse et augmentation de la cuprurie de 24 heures. Le traitement précoce basé sur les chélateurs de cuivre permet la réversibilité des lésions. Le pronostic dépend de la sévérité de la maladie lors du diagonstic et de la qualité de la prise en charge. Ce cas familial de la maladie de Wilson démontre l'importance du dépistage des membres pré symptomatique par un examen ophtalmologique et général rigoureux.

## Introduction

La maladie de Wilson est une maladie rare autosomique récessive due à une diminution de l’élimination du cuivre dans la bile et son accumulation toxique dans les organes en particulier le cerveau, le foie, l’œil et le rein d'où son hétérogénéité clinique [1]. Les manifestations ophtalmologiques représentent des critères diagnostics importants. Le traitement précoce permet une réversibilité des déficits; non traitée la maladie de Wilson est létale [2].

## Patient et observation

Deux membres d'une fratrie issus d'un mariage consanguin étaient atteints de la maladie de Wilson dans ses trois formes cliniques, hépatique, neurologique et psychiatrique.

**Figure 1:**
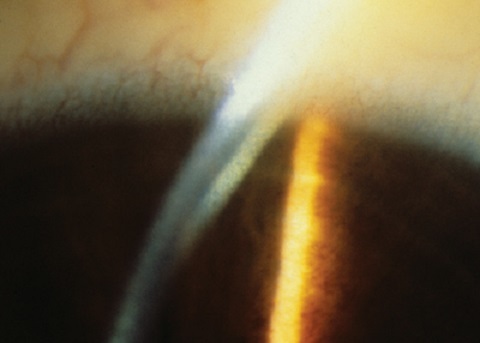
Anneau de Keyser-Fleischer à l'examen à la lampe à fente

La première âgée de onze ans présentait une maladie Wilson avec une atteinte hépatique prédominante avec un ictère associé à une ascite et une hépatite. L'examen ophtalmologique retrouvait un anneau de Keyser Fleischer bilatéral (Figure 1), le bilan biologique confirmait la maladie de Wilson en retrouvant un taux sanguin bas de ceruloplasmine à 11 mg/dl et une cuprurie de 24 heures élevée à 120 microgrammes. Le traitement était à la base de zinc per os et l’évolution était favorable.

L'aîné âgé de 14 ans présentait des symptômes neuropsychiatriques durant six mois, une labilité thymique avec des hallucinations, décédé avant toute prise en charge.

## Discussion

**Physiopathologie**: La maladie de Wilson est une maladie autosomique récessive rare s'exprimant avant 40 ans, sa prévalence est de 1 sur 30000, elle est due à la mutation du gène ATP7B porté par le chromosome 13 qui entraîne une diminution de l’élimination de l'excès de cuivre dans la bile en le liant à la céruloplasmine [3–5].

Le cuivre libre circulant est toxique car inhibe les processus enzymatiques, il s'accumule initialement au foie et provoque une dégénérescence des hépatocytes, puis passe dans la circulation et s'accumule dan d'autres organes en particulier l’œil, le cerveau et le rein [6].

La maladie de Wilson se caractérise par son hétérogénéité clinique, cependant elle se manifeste sous trois grandes formes cliniques: hépatique, neurologique et psychiatrique. Le diagnostic positif de la maladie de Wilson est fait lorsque deux de ces trois critères sont réunis: La céruloplasmine sanguine basse (normales 20 à 35 mg/dl), la présence de l'anneau de Keyser Fleischer et la cuprurie de 24 heures élevée plus de 100 microgramme/24 h chez les patients symptomatiques (normales: 20 à 50 microgramme/ 24h). Cette triade est retrouvée chez 55% des cas, il faut néanmoins suspecter la maladie même chez les patients qui ne réunissent pas tous les critères [7].

Le meilleur test diagnostic est le dosage de cuivre dans la biopsie hépatique qui retrouve plus de 200 microgramme de cuivre par gramme de tissu sec (normales: 20 à 50 microgramme/gramme) même chez les personnes pré symptomatiques.

### Les manifestations ophtalmologiques de la maladie de Wislon

L'anneau de Keyser Fleischer (FK): Décrit par deux ophtalmologistes allemands Bernhard Keyser et Bruno Fleischer. L'examen à la lampe à fente le retrouve au lime au niveau de la membrane de discret, il est mieux visible à la gonioscopie. L'anneau de KF est souvent bilatéral, sa couleur est variable (marron, jaune, bleu, verdâtre ou rougeâtre), il apparaît initialement en supérieur, puis en inférieur et devient circonférentiel et s’étend rarement plus de 5 m vers le centre. On pense que l'anneau de KF est formé par infiltration des particules de cuivre présentes dans l'humeur aqueuse à travers l'endothélium vers la membrane de descemet (Figure 1). L'anneau de KF est retrouvé chez 95% des patients atteints de la maladie de Wilson, il est également retrouvé dans 40% des cas pré symptomatiques de la maladie de Wilson. Le traitement mène à la disparition de l'anneau dans 80% à 90% des cas, sa réapparition malgré le traitement signale la non compliance au traitement.

Le diagnostic différentiel se pose avec l'anneau de Fleischer du kératocône: anneau vert ou marron à la base du kératocône, il est dû au dépôt de fer dans les cellules basales épithéliales. La cataracte en tournesol: C'est une cataracte due au dépôt de cuivre dans le cristallin. Ces manifestations ophtalmologiques apparaissent également lors de la présence de corps étrangers intraoculaires en cuivre et dans les pathologies hépatiques qui empêchent l’élimination du cuivre [8, 9].

### Les autres manifestations de la maladie de Wilson

**1. Neurologiques:** A type de dystonie, dysarthrie, dysphagie, polyneuropathie, incoordination du mouvement (parkinson like) [10]. L'IRM retrouve initialement dans les formes hépatiques prédominantes un hyper signal en T1 dans le putamen et le mésencéphale. Dans les formes neurologiques prédominantes, on retrouve un hyper signal T2 dans le striatum. Les lésions s’étendent aux noyaux gris centraux, au thalamus, au tronc cérébral et à la substance blanche. Ces lésions sont réversibles si le traitement est précoce.

**2. Psychiatriques:** Changement de la personnalité, troubles thymiques (dépression, manie, trouble bipolaire), psychose, démence sont les principales manifestations psychiatriques souvent associés aux symptômes neurologiques. L'IRM retrouve une atrophie diffuse associée à l'atteinte des noyaux gris centraux.

**3. Hépatiques et rénales:** L'atteinte hépatique et rénale conditionne le pronostic vital. L'atteinte hépatique se manifeste par une hépatite parfois fulminante, une hépatite chronique et une cirrhose pouvant nécessiter la transplantation hépatique. L'atteinte rénale se manifeste par une acidose tubulaire rénale et une diminution de la clairance de la créatinine.

**Traitement:** Le traitement est basé sur les chélateurs de cuivre: D penicillamine, trientine, climercaprol, tetrathiomolybdate et zinc. La D penicillamine a été le premier traitement oral de la maladie, cependant sa toxicité et l'aggravation des symptômes neurologiques sous traitement ont fait diminuer son utilisation. Traitement d'attaque: Manifestations neurologiques ou psychiatriques: tetrathiomolybdate. Manifestations hépatiques: trientine et zinc. Traitement des patients pré symptomatiques, de la femme enceinte et de l'enfant: zinc. Traitement d'entretien: zinc.

**Pronostic:** Le pronostic dépend de la sévérité de la maladie lors du diagnostic et de la qualité de la prise en charge. Le traitement précoce permet une réversibilité des déficits; une fois les dommages irréversibles installés l'effet du traitement est limité; non traitée la maladie est létale.

## Conclusion

Ce cas familial de la maladie de Wilson démontre l'importance du dépistage des membres pré symptomatiques par un examen ophtalmologique et général rigoureux d'où le rôle important de l'ophtalmologiste en recherchant l'anneau de Keyser Fleischer et la cataracte en tournesol.
